# Palliative Care Providers' and Administrators' Perspectives on Integrating Community-Based Palliative Care Using the Social Determinants of Health Framework

**DOI:** 10.1089/pmr.2023.0048

**Published:** 2024-02-05

**Authors:** Savannah Horvick, Nancy Dias

**Affiliations:** Department of Nursing Science, College of Nursing, East Carolina University, Greenville, North Carolina, USA.

**Keywords:** community-based palliative care, social determinants of health, palliative care policy, access to resources, institutional buy-in

## Abstract

The purpose of the study is to examine the perspectives of palliative care (PC) providers about the needs, barriers, and disparities to integrate PC into the community setting. This study used a descriptive qualitative design to complete a phone interview that incorporated the United States Department of Health and Human Services' (USDHHS) social determinants of health (SDOH) framework domains, as well as a demographic survey. Thirteen PC providers and administrators were interviewed to ascertained their perceptions about community-based palliative care (CBPC) related to the SDOH. Subthemes were ascertained using the USDHHs' SDOH as themes: lack of patient access to resources, challenges of institutional philosophic buy-in, gaps in medical education, lack of health care literacy among patients, distrust in health care, differing perspectives on quality of life, social and community support, environmental injustice, lack of interdisciplinary collaboration, multidisciplinary continuing education, and CBPC program evaluation. The results from this study provide evidence to help identify potential barriers to planning necessary CBPC interventions based on potential SDOH disparities.

## Introduction

Palliative care (PC) is a specialized segment of medicine that aids those with serious or chronic illnesses by focusing on their quality of life, as opposed to diagnosis and disease prognosis. PC is beneficial at any stage of a disease process and will support the medical, social, emotional, and practical needs of a patient either in a hospital, clinic, or at home. This care can be combined with curative care, while still emphasizing comfort.^1^ Community-based palliative care (CBPC) provides local resources and guidance for the providers and community health nurses working with each patient to ensure multidisciplinary care and empathetic communication.^2^ Worldwide, only 14% of the people who need PC receive that care.^3^

CBPC emulates many aspects of primary PC and their standards of practice; however, it has been found that CBPC's flexible approach allows better access and communication than primary PC. Proper funding and access to resources is a challenge for this less established method of PC delivery.^2^ It has been demonstrated through prior research that to provide effective evidence-based CBPC, providers must understand the social determinants of health (SDOH) of the population they are caring for.^4,5^ There is currently ample information about the implementation of CBPC and efficacy; however, there is not a study that interprets the barriers to CBPC through the lens of the SDOH.^5^

## Objectives

The purpose of the study was to understand the needs, barriers, and disparities to implementing CBPC through the lens of SDOH.

### Design

Convenience and snowball sampling was used to enroll participants in this study. The semistructured telephone interviews were recorded and transcribed verbatim. All transcriptions were deidentified before analysis occurred. Data were analyzed using the directed content analysis from Hsieh and Shannon's conventional content analysis framework and manual qualitative analysis, guided by the SDOH framework.^5,6^

### Settings/subjects

Ten administrators and three providers (medical doctor, nurse practitioner) with minimally 1 year of experience in PC and individuals >18 years old participated in phone interviews regarding CBPC.^7^ The participants represented seven different states within the United States.

## Results

PC providers whose primary role was administrative made up most of the sample size, with all of the interviewees having graduate education (see Table 1 for demographic information).

**Table 1.** Demographic Information of 13 Professionals Interviewed

Variable	Value	*N* * = 13, * *n* * (%)*
Gender	Male	5 (38.4)
	Female	8 (61.5)
Age (years)	30–49	5 (38.4)
	50–64	6 (46.1)
	65+	2 (15.3)
Race	White	10 (76.9)
	Black or African American	2 (15.3)
	Prefer not to answer	1 (7.6)
Highest level of education	Unspecified graduate school	1 (7.6)
	Masters	5 (38.4)
	Postmasters	1 (7.6)
	PhD	3 (23.0)
	MD	3 (23.0)
Certification(s) in palliative care	Yes	10 (76.9)
	No	3 (23.0)
Current positions	Administrator	10 (76.9)
	Provider	3 (23.0)

The themes highlighted the needs, barriers, and disparities in providing CBPC through the lens of the SDOH. The five SDOH and their subthemes were used to represent the data. The themes and subthemes are illustrated in Table 2.

**Table 2.** Five Social Determinants of Health Themes and 11 Subthemes from Telephone Interviews With Palliative Care Professionals

Themes	Subthemes	*Occurrences,^a^* *n* * (%)*	Definitions	Quotes
Economic stability	Lack of patient access to resources	54 (6.3)	Patient access to resources such as Internet, time-off work, transportation, stable housing, and medication	“They need to work a certain amount of time to qualify for FMLA?… employers sometimes really discourage them from taking prolonged breaks”
	Challenges of institutional philosophic buy-in	92 (10.8)	Institutional financial support related to the cost-reduction strategy of palliative care	“They created their own insurance plan… help shape service delivery from within”
Education access and quality	Gaps in medical education	104 (12.3)	Lack of education related to palliative care throughout medical education	“Have someone come in and talk about legal documents, like an advance and a living will and how important those are to teach”
	Lack of health care literacy among patients	49 (5.7)	Lack of health care education related to palliative care	“I hear at least once a week… ‘Wow, you're the first person and that's been able to help me understand this’”
Social and community context	Distrust in health care	39 (4.5)	Distrust in the health care system related to underserved populations	“There's a distrust in general of the health system…Most of these patients have been through the ringer of healthcare”
	Differing perspectives on quality of life	78 (9.2)	Patient and provider perspectives on quality of life and what defines quality of life	“For our patients of color, losing the support of hospice and end of life in disproportionate numbers… and have a very poor quality and end of life experience”
	Social and community support	17 (1.9)	Support and resources available for patients to assist in palliative care delivery	“I tried to have a patient fill out a form for his inhalers…Well, they didn't have it in the Spanish form and this patient spoke Spanish”
Neighborhood and built environment	Environmental injustice	106 (12.5)	Environmental factors that disproportionately impact underserved populations including food deserts, geographic location, and infrastructure	“Oftentimes there are no sidewalks in the communities that I'm going into… telling people to exercise, it may not be safe for them to go out and walk in their neighborhoods”
Health care access and quality	Lack of interdisciplinary collaboration	134 (15.9)	Processes in palliative care that can be improved through interdisciplinary collaboration including referrals, assessment tools, and concurrent care	“It [CBPC] needs… a social worker, a chaplain, a physician, nurse, nurse practitioners”
	Multidisciplinary continuing education	94 (11.1)	Methods and topics to be included in palliative care continuing education including nursing scope of practice, substance abuse, telehealth, spiritual care, care delivery models, and standardized patients	“Using standardized patients… the patient is trained to get frustrated or angry if the provider doesn't address their emotions properly, does not communicate information without using medical jargon”
	CBPC program evaluation	59 (6.9)	Measures that can be used to indicate the quality of health care being delivered including staff retention, staff diversity, and access to care	“I think the barrier to accessing PC is one, are the services available in their area?”

^a^ Occurrences reflect the number of times that codes under this subtheme were identified in interview transcripts.

CBPC, community-based palliative care.

### SDOH Theme 1: Economic stability

The subthemes below describe how providers and administrators strive to create economic stability within CBPC for patients and health care entities.

#### Lack of patient access to resources

Resource accessibility was a barrier for patients to both access and maintain this type of care. Resources included Internet, time-off, transportation, housing stability, translated documents, and medication costs. Accessibility to these resources is necessary to sustain effective CBPC programs.

#### Challenges of institutional philosophic buy-in

Providers and administrators of PC endorsed the difficulties of creating and maintaining a CBPC program, due to the financial constraints. CBPC reduces health care costs in the long term; however, administrators stated that it does not create profit in the short term. Therefore, institutions must agree with the philosophy behind these programs to create and maintain them. This barrier can be overcome by an evaluation of insurance coverage and coding by an independent interdisciplinary council that will incentivize PC.

### SDOH Theme 2: Education access and quality

This subtheme describes the gaps in basic PC education within health care institutions, as well as in the public, that shape current CBPC delivery.

#### Gaps in medical education

Throughout the interviews, it was highlighted that medical education focuses on skills, but conversation is not viewed as a skill. Therefore, providers are not trained in conducting PC conversations, such as advanced care planning, disease prognosis, and end-of-life care planning. Curricula changes were recommended to include conversation skill training in medical education. In addition, training about how and when CBPC referrals need to be included in the curriculum for all providers, so that early referrals to CBPC can be made. Conversation skill development and PC education are vital to shaping the future of CBPC delivery.

#### Lack of health care literacy among patients

Health care illiteracy is a pervasive issue in all medical specialties, as with PC. It was explained that for a patient to make proper decisions about their illness, they must be properly educated on the illness, its prognosis, and the role of CBPC. Health care illiteracy creates a barrier and disparity for patients to access CBPC; however, caregiver dashboards, PC reference books, and social marketing are proposed solutions to address CBPC health care illiteracy.

### SDOH Theme 3: Social and community context

The subthemes describe the various factors within a patient's surrounding community that have an impact on their CBPC.

#### Distrust in health care

Throughout the interviews, it was reported that there was a distrust in the health care system, namely within minority and/or underserved populations. Providers expressed that minority, specifically African American, patients have a distrust of the health care system, due to the history of racial discrimination, both as a country and within the health care system. Health care professionals have a duty to be advocates for their patients to build patient–provider trust. Advocacy could mitigate the distrust in health care that currently exists.

#### Differing perspectives on quality of life

Both patients and providers have unique definitions of quality of life that impact a patient's PC. A patient's quality of life can be defined by their advanced care planning, religion, culture, perspective on PC, reluctance to reach the end of their life, their social debt, and their caregiver support. For example, if patients' quality of life is defined by their religion, while their CBPC provider is focusing on advanced care planning, there is a gap in care created by the misaligned perspective on quality of life. The provider and the patient should formulate a mutually agreed upon CBPC plan to address potential discrepancies.

#### Social and community support

A vital component of CBPC is the social and community support that patients have around them. This can be as simple as having access to programs at church, family members who can act as caregivers, and supportive programs by local government institutions. Without proper social and community support, patients cannot fully reap the benefits of CBPC.

### SDOH Theme 4: Neighborhood and built environment

The subtheme describes the disproportionate impact that the environment has on underserved populations.

#### Environmental injustice

Besides simple geographic access to CBPC programs, patients struggle to access resources such as food and infrastructure, due to the neighborhood where they reside. There are discrepancies in health care availability and care settings, which need to be addressed through economic development for affected locations. When an area has a lack of economic development, it impacts the amount of health care professionals who are attracted to that region. The SDOH framework can be used as an assessment tool to assess the needs, barriers, and disparities to provide CBPC.

### SDOH Theme 5: Health care access and quality

The subthemes detail the factors and proposed solutions that can improve CBPC access and quality.

#### Lack of interdisciplinary collaboration

Referrals, assessment tools, and concurrent care are vital parts of the CBPC model that both require and promote interdisciplinary collaboration. To create a strong CBPC program, it requires the collaboration of health care professionals to provide early seamless referrals from acute and primary care to increase the continuity of care between medical specialties, which allows for more comprehensive grief support. Opportunities for continuing education for health care professionals on the methods of interdisciplinary collaborations and the benefits it provides need to be integrated within health care systems.

#### Multidisciplinary continuing education

Practicing providers can further their education through continuing education programs. The interviews suggested that covering topics such as nursing scope of practice, substance abuse, telehealth delivery, spiritual care, and care delivery models could improve CBPC delivery. Standardized patients, registered nurse fellowship programs, and other unique educational models are reported to provide higher quality education to providers.

#### CBPC program evaluation

Continuous evaluation of PC programs, alongside measurements of health care quality, can be beneficial to improving and evaluating the impact of a program. These indicators can include staff retention, staff diversity, and access to care. Quality indicators can help evaluate program performance to create and sustain effective CBPC programs.

## Conclusions

CBPC, when informed by the SDOH, is an evidence-based care delivery model that expands the utilization of PC and increase the efficacy of care provided.^4,5^ The results from this study identify barriers, needs, and disparities to providing CBPC and necessary changes in medical education, health care policy, and clinical practice to improve CBPC. There is a need for increased education of the public and medical professionals about what CBPC is and how it can support patients, but this begins with providers having a more comprehensive PC education within their medical school.

Medical education should include skills such as leading goals-of-care conversation, advanced care planning, and symptom management, as well as educating providers on when PC referrals are appropriate.^8^ Recommended CBPC policy changes should include support for concurrent care, incentivize CBPC in insurance coverage, and increase billable skills for PC.^9^ In terms of clinical practice, the development of a needs assessment tool based on the SDOH framework would help the multidisciplinary care team provide more holistic care to CBPC patients by effectively ascertaining the needs of each patient.

In addition, CBPC-specific quality indicators should be utilized to evaluate CBPC programs, which can include hospital admission data, symptom and pain management, place of death, prevalence of advance directives, and caregiver distress.^10,11^ The SDOH framework can be an effective framework to assess patient needs, promote and delivery interdisciplinary care, and evaluate CBPC program quality.

CBPC, when informed by the SDOH, is an evidence-based care delivery model. However, this care delivery model has systemic and hospital-based challenges that prevent CBPC from being as effective as possible. The results from this study provide foundational evidence to help identify potential barriers to planning necessary CBPC interventions that are based on SDOH disparities. This study informed educational concepts, practices, and policies that may improve PC. A systemic review of transitions to CBPC found that there is a need for research to transition patients more efficiently and assist clinicians in this transition.^12^

This is reflected in the results from this study—care settings, referrals, and care delivery models are codes with connected quotes that reflect the systemic review's findings. From this study, further research is necessary. An assessment tool using the SDOH framework would help the multidisciplinary team provide more holistic care to patients. There is a need for PC policy changes related to accessibility, insurance coverage, concurrent care, and PC billing to promote CBPC.

## Abbreviations Used

CBPC: community-based palliative care
PC: palliative care
SDOH: social determinants of health
USDHHS: United States Department of Health and Human Services’
